# A Case Study of Performance Degradation Attributable to Run-Time Bounds Checks on C++ Vector Access

**DOI:** 10.6028/jres.118.012

**Published:** 2013-05-22

**Authors:** David Flater, William F Guthrie

**Affiliations:** National Institute of Standards and Technology, Gaithersburg, MD 20899

**Keywords:** bounds checking, buffer overflow, C, C++, performance, security

## Abstract

Programmers routinely omit run-time safety checks from applications because they assume that these safety checks would degrade performance. The simplest example is the use of arrays or array-like data structures that do not enforce the constraint that indices must be within bounds. This report documents an attempt to measure the performance penalty incurred by two different implementations of bounds-checking in C and C++ using a simple benchmark and a desktop PC with a modern superscalar CPU. The benchmark consisted of a loop that wrote to array elements in sequential order. With this configuration, relative to the best performance observed for any access method in C or C++, mean degradation of only (0.881 ± 0.009) % was measured for a standard bounds-checking access method in C++. This case study showed the need for further work to develop and refine measurement methods and to perform more comparisons of this type. Comparisons across different use cases, configurations, programming languages, and environments are needed to determine under what circumstances (if any) the performance advantage of unchecked access is actually sufficient to outweigh the negative consequences for security and software quality.

## 1. Introduction

The run-time environments of many programming languages, including Java,^1^ Visual Basic, and numerous interpreted scripting languages, will throw an exception or abort the program any time that an invalid array access is attempted. This does not occur for the C language, nor for C arrays that are used in C++ or Objective-C. The C++ language offers a safe access method in the Standard Template Library’s vector class, but idiomatic usage instead invokes an operator[] function that is no safer than C array subscripting [1]. NSArray, an analogous *de facto* standard library class for Objective-C, provides only a safe access method [2]. The environments for C++ and Ada normally enforce safety conditions, but the programmer can disable safety checks using pragmas and compiler switches. In C++, one uses unsafe methods of array access within declared unsafe regions [3]; in Ada, one uses the Suppress Index_Check pragma [4].^2^

As of March 2013, C and C++ are ranked second and fourth in the Programming Community Index published by TIOBE Software [6]. Java and Objective-C are ranked first and third respectively, with Java being only 1.015 % more popular than C. For the purposes of this report, it suffices to establish that much new development continues to be done in C and C++; the reasons for their continued high ranking are fuel for a separate discussion.

As documented in CWE-129 of the Common Weakness Enumeration [7], the common consequences of improper validation of array indices include serious degradations of security, such as the possibility of an attacker executing arbitrary code, and of software quality, such as data corruption resulting in incorrect program behavior. Despite the opportunity to prevent these consequences, programmers routinely omit run-time safety checks from applications because they assume that these safety checks would degrade performance.

This report documents an attempt to measure the performance penalty incurred by two different implementations of bounds-checking in C and C++ using a simple benchmark and a desktop PC with a modern superscalar CPU. Each of 12 different coding constructs, called *variants*, was compiled using a range of different compiler options for levels of optimization and optional bounds-checking. The average CPU time used by each combination of variant and compiler options was measured. The combination that exhibited the best performance for bounds-checked access and one of several combinations that exhibited the best performance observed regardless of bounds checking were then compared, taking care to account for the effects of multiple comparisons that arise in statistical analyses [8,9].

In addition to the results of that comparison, significant findings were that further work is needed to develop and refine measurement methods and to perform more comparisons of this type—for example, to compare the performance of bounds-checked C++ code with the performance of equivalent Java code for various use cases in which safety checking matters. Comparisons across the different dimensions of use cases, hardware configurations, programming languages, and run-time environments are needed to determine under what circumstances (if any) the performance advantage of unchecked access is actually sufficient to outweigh the negative consequences for security and software quality.

The remainder of this report is organized as follows. Section 2 discusses related work, including alternatives to run-time bounds checking and methodical approaches to software performance measurement. Section 3 provides the precursory technical information necessary to define the experiment that was conducted. Section 4 provides the post-experimental summary and analysis, including the performance comparison that was the ultimate goal. Section 5 describes follow-on work, and Sec. 6 concludes.

## 2. Related Work

The related work includes the two disparate areas of research and practice that are discussed in the following two sections, namely, methods of enforcing safe access to array data and methods of measuring software performance.

### 2.1 Alternatives to Run-Time Bounds Checking

The bounds-checking methods explored in this case study operate at run time. An alternative or complementary technique that can be used in conjunction with run-time checking of array bounds is static analysis. Static analysis tools [10] can identify potential violations in code that has not yet executed, thus providing superior coverage for applications that are impossible to test exhaustively. However, the static analysis approach is inherently more complex and challenging to implement, and it is vulnerable to both false positives and false negatives when identifying potential violations.

Another alternative to checking array indices at the application level is to allocate additional pages of memory in such a way that an attempt to index beyond the end of an array will trigger a segmentation fault [11,12]. The efficiency and effectiveness of this approach is limited by the granularity of pages and alignment constraints, and less common errors that result in the access of a valid-but-wrong portion of memory (e.g., a different array than the one that was intended), completely missing the additional allocated pages, would not trigger a segmentation fault.

Finally, in C++, one can employ template metaprogramming techniques [13] to draft the compiler into verifying safety preconditions at compile time without incurring any run-time overhead. Since one is limited to assertions that are decidable at compile time, this idea could be characterized as a different implementation of static analysis; as such, it has limitations similar to static analysis using external tools.

### 2.2 Benchmarking

In the computing context, performance measurements, whether absolute or relative, are seldom accompanied by methodical treatments of measurement uncertainty and variability of the sorts that were encountered in this case study. Although the increasing complexity of computer systems makes measurement science ever more important, Berry’s observation in 1992 [14] that statistical design of experiments had not often been applied to the analysis of computer system performance remains true today. Notable exceptions include Refs. [15] and [16] and examples surveyed in Ref. [17]. Complications encountered in the present case study justify continued work on the measurement issues and a more thorough synthesis of computer benchmarking practices with experimental design and analysis.

Tools for software performance measurement are informally divided between those designed for “micro-benchmarks” of individual instructions or short code blocks viewed in isolation and those designed for application-level performance profiling. The measurements done in this case study would be in the micro-benchmark category. Some resources and test programs useful for micro-benchmarks on x86 platforms are included in or cited by Dr. Agner Fog’s collection of software optimization resources [18]. Those test programs use inline assembly and a specialized kernel driver to access hardware counters, whereas this case study used an existing high-level API (clock_gettime [19]). Two other APIs providing access to the hardware counters are the Linux performance counters subsystem, also known as the perf_events subsystem [20], and the Performance Application Programming Interface (PAPI) [21] that is layered above it [22]. Both of these APIs can be used to implement either application profiling tools or micro-benchmarks. The more powerful application profiling toolsets, such as the perf tools [23] that are bundled with the Linux kernel, are capable of resolving results down to the instruction or code block level of granularity, but their use of interrupt-driven sampling introduces an uncertainty component that can be avoided in micro-benchmarks.

Studies that directly evaluate the properties of the hardware performance counters themselves are rare but informative. Dmitrijs Zaparanuks et al. [24] noted the significant variability of cycle counts which reproduced in this research. Variability and error in results derived from instruction counts have been explored by Dr. Vincent M. Weaver and associates [25-27].

Many benchmarks exist for comparing the performance of different hardware configurations, rather than software performance; see Ref. [28] for an example benchmark suite and for links to more related work.

## 3. Preliminaries

This section provides the precursory technical information necessary to define the experiment that was conducted, including definitions of terms, implementation details, controlled and independent variables, and specification of the methods used.

### 3.1 Terms

The following terms are used throughout the remainder of this paper.

**Table:** 

Variant	Particular coding construct for array access.
Benchmark	Code segment that outputs execution time data for a given variant.
Datum	Number (representing CPU time) printed by a single iteration of a benchmark. A single iteration yields two data: one for the “small” array, one for the “big” array.
run	One execution of a program. In a given run, each benchmark in the program is executed many times.
series	All data printed by a given benchmark within a single run for a specified array size. A single run yields one series for the “small” array size and one series for the “large” array size for each benchmark in the program.
series mean, etc.	Statistics over all the data of a particular series.
cell	All series with the same optimization and variant for a specified array size.
cell mean, etc.	Statistics over all data of all series in a cell, without regard to which series they came from.

### 3.2 Bounds-Checking Implementations

The case study investigated two different implementations of run-time bounds-checking:

1. The at() member function of the C++ Standard Template Library’s vector class is a simple accessor function that throws an exception if the index is out of range. It is a substitute for the operator[] function, which provides the idiomatic array access syntax but no assurance that the index will be validated before use.
2. Mudflap [29,30] is a more general run-time checking framework for C and “very simple” C++ programs that is integrated with the GNU Compiler Collection (GCC). Mudflap instruments all risky pointer/array dereferencing operations and a variety of other risky constructs. It does not require changes to source code, but it does require particular options at compile and link time, a run-time library, and run-time configuration through environment variables. With appropriate configuration, one can enable run-time checking for all array accesses or for only write operations. It is also possible to build Mudflap support into an executable but selectively enable or disable checking at run time. Although Mudflap can be enabled for C++ language programs, there is a history of spurious violations with that combination [31], so for this study it was used only with C.

Neither of these run-time checking implementations introduces any additional static, compile-time checking.

## 3.3 Benchmarks and Timing

**Fig. 1:**
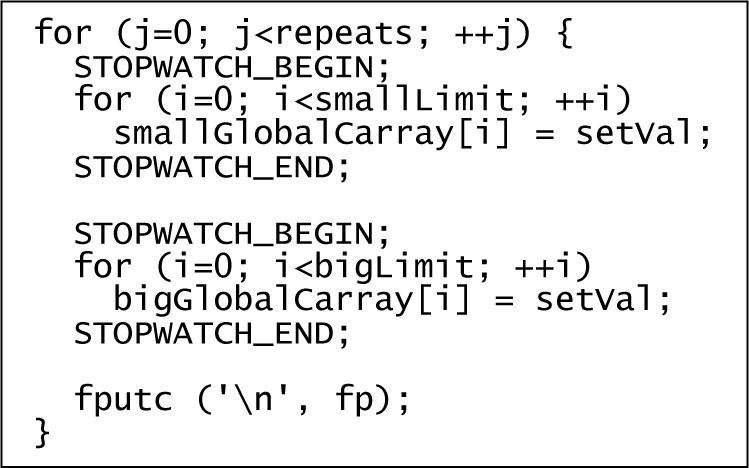
Example CPU time measurement loop.

The C/C++ code example in Fig. 1 shows how the CPU time used to execute a variety of methods of assigning to elements of an array or array-like data structure was measured. The inner statements of the loop were replaced as appropriate to each measurement.

Two different sizes of arrays were used to enable a comparison that would reveal the magnitude of constant time overhead that was included in results, and also to reveal if and when the assignment loops were eliminated by optimization (as the results would then become independent of the array size).

The number of iterations for the inner statements was fixed (smallLimit and bigLimit, respectively), and the CPU time required to execute that number of iterations was measured. An alternate approach is to fix an amount of time and measure the number of iterations that are completed during that time interval [32]. In this case, fixing the number of iterations was more straightforward.

The parameters used were repeats = 2000, smallLimit = 10^6^, and bigLimit = 10^7^. Arrays were declared as global variables to avoid dead variable elimination. The element type was int32_t.

The STOPWATCH_BEGIN macro stored the return value and timespec returned by clock_gettime (CLOCK_PROCESS_CPUTIME_ID, …). The STOPWATCH_END macro did this again, and then invoked a function to check the return values and print out the difference of the returned timespecs in nanoseconds. Thus, in the absence of instruction reordering by the compiler or CPU, the printed value would include the quantity of interest plus the overhead of invoking clock_gettime and storing its returns once.

The clock CLOCK_PROCESS_CPUTIME_ID is supposed to reflect CPU time used by the process, not real time. It is traceable to a high-resolution hardware counter and claims 1 ns resolution (according to clock_getres). The precision and accuracy of the underlying counter and the derived CPU time clock were not verified empirically, but the distribution of values returned by clock_gettime showed recognizable structure on a microsecond scale, demonstrating adequate resolution for this work.

It has been documented that clock_gettime can return “bogus results” when a process is migrated from one CPU to another, as the CPUs’ clocks are not synchronized [19]. To prevent this, the processes were locked onto CPU 0 using the libc function sched_setaffinity.

### 3.4 Controlled Variables

Tests were run on a Dell PC running Slackware Linux with the compiler and kernel upgraded to newer versions than were included with the Slackware distribution. Details are provided in Table 1.

The kernel configuration was not necessarily optimal for the task at hand, but an investigation of the impact of changing kernel configurables was deferred to future work.

Other variables were controlled as follows:

- Data caching: Since the benchmark linearly traversed a data set that was larger than the L2 cache, alternating between the small and big arrays, one would expect consistent behavior from the data cache over time.
- Instruction caching: For runs of executables built without Mudflap support, the compactness and iterative nature of the benchmarks should have sufficed to minimize the occurrence of instruction cache misses that were inherent to the benchmarks. Instruction cache misses after the first iteration were still possible if other processes or the kernel made significant use of the shared cache. For Mudflap runs, an unknown quantity of additional run-time library code was involved, so this variable was not controlled.
- CPU / timer frequency: The CPU did not support Turbo Boost, the CPU frequency scaling feature was excluded from the Linux kernel (the feature was not compiled), and the SpeedStep feature was turned off in BIOS settings. It was not possible to exclude the generic CPU idle infrastructure because of kernel dependencies. With the configuration chosen, “none” was the driver reported by/sys/devices/system/cpu/cpuidle/current_driver. Data collection began with hardware components at warmed-up, idle operating temperature and ran to completion without interruption.
- Unanticipated multithreading (e.g., transparent parallelization of Standard Template Library operations): Creation of additional threads was prevented by setting RLIMIT_NPROC to 0. No parallelizing functions were knowingly used.
- Invalidation of results due to instruction reordering or code elimination by the compiler: While it was not done for every benchmark and optimization level, the compiled code that was used in the final performance comparison was disassembled to demonstrate that the results were not invalidated by the compilation.
- Overhead of measurement: The overhead of the symmetrical recordings of the value of the clock that occurred at the start and end of a timed run was included consistently in every measurement. The magnitude of the constant time overhead was evaluated by comparing the results from benchmarks run with two different sizes of arrays.

One factor that may not have been controlled is instruction reordering at the CPU (micro-op) level causing instructions to effectively move into or out of the timed region. The Linux kernel implementation of the syscall that clock_gettime invokes retrieves values that are traceable to the Time Stamp Counter via the process scheduler. Although steps are taken in the code to stabilize the results, the authors have not yet located an invocation of a serializing instruction such as cpuid in the path of the syscall.

### 3.5 Complications

Initial exploration suggested that the following potential complications should be addressed. Plots and tables to illustrate these complications have been omitted for brevity.

- The first datum of a series may be an outlier. However, while it often is the maximum or minimum datum in the series, it remains in the same order of magnitude as the mean.
- The series from the first benchmark executed in a run may have greater variance than the series from subsequent benchmarks in the same run. In some cases, scatter plots of series data vs. iteration number revealed a “stabilization period” during which the mean performance improved and then leveled off. In other cases, the first datum of the series was a more extreme outlier.
- The difference between the means of two series within a cell can be of a greater magnitude than the variance of each of those series figured independently. This suggests, unsurprisingly, that the performance of a benchmark is affected by environmental factors that can change from one run to the next. This effect will be referred to as *inter-process variation*.
- Results can be significantly affected by competing loads (other processes) running on the same CPU.
- Results can show time-dependency in the form of abrupt changes that are apparent in scatter plots of series data vs. iteration number. These effects range from insignificant changes in the distribution of “long tail” outliers (changing the shape of the thin, scattered cloud above the main distribution), to transient spikes, to significant migration of the entire distribution.

These complications were addressed as follows:

- Since every loop in real life has a first iteration whose performance is relevant, and since the outliers were not extreme enough to move the mean significantly, nothing special was done about the initial outliers. If it were desirable to exclude them, they could simply have been dropped, or the median could have been used instead of the mean.
- The benchmarks within runs were rotated in a Latin-square-like design so that each benchmark within a given program would be affected equally by the first-benchmark effect. The data from the first benchmark in each run were retained for the same reasons that the initial outliers were kept: in real life, something has to go first, and the resulting noise was not strong enough to bury the signal of interest.
- Data were collected over a larger number of runs, rather than a few long runs, so that the presence or absence of inter-process variation would become evident.
- Data were collected with the operating system in single-user mode, eliminating unnecessary processes, but at normal scheduling priority.
- To test for the presence of significant time-dependency, as well as inter-process variation, the Durbin-Watson statistic [33, 34] was calculated over the chronologically ordered concatenation of all series within a cell. (All data and all series were kept in chronological order.)

### 3.6 Independent Variables

The variants are listed in Table 2. The operations tested included three using C arrays or pointers that were compiled as both C and C++ and six using std::vector for C++ only, for a total of twelve variants split across two programs.

The language dialects used were C99 and C++11 as selected by the GCC command-line options –std=c99 and –std=c++0x. Both programs were compiled with –D_GNU_SOURCE in order to access the functions necessary to lock the running thread onto CPU 0.

Some of the variants used a level of indirection, in the form of an array of indices that was read from an external file at the start of the program, to obscure indices from compile-time analysis. In each case, the value of indices[i] was i, but that fact could not be determined at compile time.

Benchmarks were executed in Latin-square-like rotations. At each rotation, the benchmark that was previously first to execute in a program was moved to the end of the list, but the order of the benchmarks was not otherwise shuffled. For the C++ program, each of the 9 benchmarks was in the first position 1/9 of the time; for the C program, each of the 3 benchmarks was in the first position 1/3 of the time. (Consequently, if the “stabilization period” effect were significant, one would expect to see greater variance in the C results.)

Those rotations, it turn, were multiplied by a similar rotation of 7 “optimization levels.” 4 of the 7 were, in fact, the GCC-defined optimization levels –O0 through –O3, with –O0 indicating no optimization and –O3 indicating the maximum generally safe optimization. The other 3 combined additional options with –O3:

–flto enables link-time optimization, which is an additional layer of optimization that can actually be combined with any optimization level –O0 through –O3. The combination –O3 –flto was designated “O4.”
–fmudflap –fmudflapir enables the Mudflap run-time checking option of GCC with the ignore-reads behavior in effect. The C program was compiled with the options –fmudflap and –fmudflapir and executed with MUDFLAP_OPTIONS=‘–mode-check –viol-abort –ignore-reads’. The combination –O3 –fmudflap –fmudflapir was designated “O5.”
–fmudflap enables Mudflap without the ignore-reads behavior. The C program was compiled with the option –fmudflap and executed with MUDFLAP_OPTIONS=‘–mode-check –viol-abort’. The combination –O3 –fmudflap was designated “O6.”

The resulting executables with their sizes are listed in Table 3. All of the bench_C executables were built from the same source file and implemented benchmarks for all three of the C language variants. Similarly, all of the bench_C++ executables were built from the same source file and implemented benchmarks for all nine of the C++ language variants.

As was mentioned in Sec. 3.2, compiling the C++ program with Mudflap was not attempted because of a history of spurious violations with that combination [31]; hence, there is no bench_C++_O5 or bench_C++_O6 executable. Similarly, –flto could not be combined with –fmudflap because it resulted in “lto1: internal compiler error: mudflap: this language is not supported.”

A utility module containing overhead functions that were used only outside of timed segments was compiled only once, at –O3, and linked with each program.

### 3.7 Verification of Bounds Check Effectiveness

Out-of-bounds assignments were attempted immediately prior to each program’s exit to collect evidence that safety conditions were or were not being enforced. To avoid unhealthy side-effects, the out-of-bounds assignments that were expected to be allowed were performed last.

**Fig. 2:**
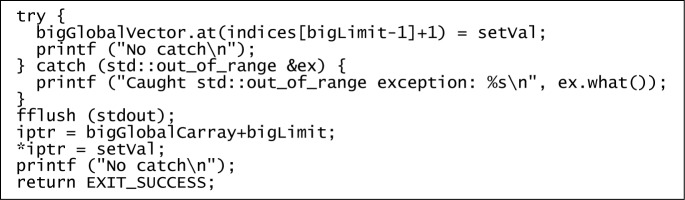
Verification of bounds check effectiveness.

The end of the C++ program is quoted in Fig. 2. The end of the C program has only the pointer assignment portion.

## 4. Data and Results

This section provides the post-experimental summary and analysis of data collected, makes the performance comparison that was the ultimate goal of the experiment, provides disassemblies of the critical code segments to exclude unexpected compiler behavior as a factor, and discusses limits on the generality of the results.

### 4.1 Data Collection

The rotations were driven by a Bash script. The rotation of benchmarks within programs was accomplished by passing a command-line argument to the main program, which then invoked benchmarks in the order indicated. In total, with 9 rotations of benchmarks, 7 rotations of optimization levels, and 2000 data per series, each cell accumulated 63 series and 126 000 data from 63 different runs of the same executable.

Table 4 gives the cell means for the “big” arrays (10^7^ elements) with variants appearing as columns and optimization levels as rows. The number following the symbol ± is the numerical value of an expanded uncertainty for each cell mean, *U*, which is defined below. Cells where the Durbin-Watson statistic calculated over the chronologically ordered concatenation of all series was less than 1, indicating significant time-dependency in the data and consequently engendering less trust in the results, are shown in italics.

In Table 4, the expanded uncertainty *U* = *ku* was determined from an estimated standard uncertainty (estimated standard deviation of the cell mean) *u* and a coverage factor *k* = 4.31. Because of inter-process variation, the usual standard uncertainty of the cell mean [35] for a sample size of 126 000 would have been overly optimistic. Instead, *u* was calculated as the experimental standard deviation of the mean of the series means. Letting *X_k_* represent the series means and $\bar{X}$ the cell mean, for *n* = 63 runs,

$$u={\left(\frac{1}{n(n-1)}\sum_{k=1}^{n}{\left(X_{k}-\bar{X}\right)}^{2}\right)}^{\frac{1}{2}}.$$

The value of the coverage factor was chosen to control the overall confidence level of all different assessments or comparisons of interest for this analysis. This was achieved by using the Šidák inequality [8,9] to control the individual confidence levels of intervals for each cell mean (of which there are 66) and of all potential pairwise comparisons of an unsafe array access to a safe alternative (of which there are 50 × 16 = 800), for a total of 866 simultaneous statements. Using this approach, the confidence level for each individual statement is taken to be 0.95^(1/866)^ = 0.9999408. The value of *k* for each cell mean uncertainty was computed from the Student’s *t*-distribution with *n* – 1 = 62 degrees of freedom. The value of *k* for pairwise comparisons was also computed from the Student’s *t*-distribution, but with degrees of freedom obtained using the Welch-Sattherthwaite formula [36].

The simultaneous control of the confidence of all uncertainty statements of interest yields 95 % confidence that *all* of the statements are correct. This mitigates the concern that the choice of best-performing cells for comparison could introduce bias through a form of “cherry picking.” If it were instead the case that each individual mean or pairwise comparison had a 5 % risk of being outside its stated interval, looking at multiple means and choosing the best-performing cells for comparison could amplify the risk of reaching incorrect conclusions to greater than 5 %.

Table 5 gives the Durbin-Watson statistics for each cell as a measure of time-dependency and inter-process variation, which manifest as apparent autocorrelation in the cell’s data.

Table 6 gives the ratios of the respective cell means for the “big” and “small” arrays. Detailed data from the “small” arrays are otherwise omitted for brevity.

Table 7 gives the ratio of the experimental standard deviation of the mean of series means to the experimental standard deviation of the cell mean as it would be computed if all 126 000 data points in the cell were independent measurements. Values of this ratio significantly larger than unity suggest the presence of inter-process variation and support the decision to assess the standard uncertainty of each cell mean using the 63 series means rather than the individual data points. No other correction was made for any apparent autocorrelation in the data.

Since it can be assumed by the Central Limit Theorem that the observed values of the cell means are approximately normally distributed with approximate standard deviation *u*, for cells not exhibiting excessive time-dependency, the unknown true cell mean values are believed to lie in the intervals defined by $\bar{X}\pm U$ with a simultaneous level of confidence of at least 95 %. The assumption of approximate normality of the cell means was confirmed using normal probability plots of the sample means of bootstrap samples drawn from the empirical distribution of series means within each cell. Potential biases in the bootstrap statistics were compared to the corresponding statistics actually observed using histograms of the bootstrap statistics and no significant biases were identified.

The outcomes of the run-time tests for effectiveness of safety checks are shown in Table 8. All were as expected. “No catch” means that the “No catch” message was printed and the program exited with no error messages, segmentation faults, or other abnormal behavior observed.

The invalid assignments through pointers were coded so blatantly as to trigger a compile-time “warning: array subscript is above array bounds” from static analysis at optimization levels −O2 and higher, yet they apparently succeeded at run time except when Mudflap was active. The invalid assignments through vector.at() were obfuscated with indirection, but they were still caught at run time.

### 4.2 Analysis

**Fig. 3:**
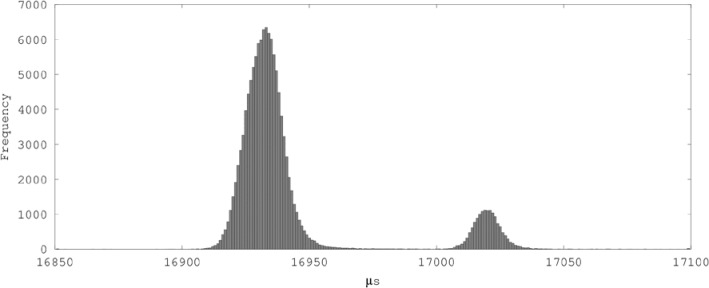
Histogram of cell data for vector[i] at –O3.

**Fig. 4:**
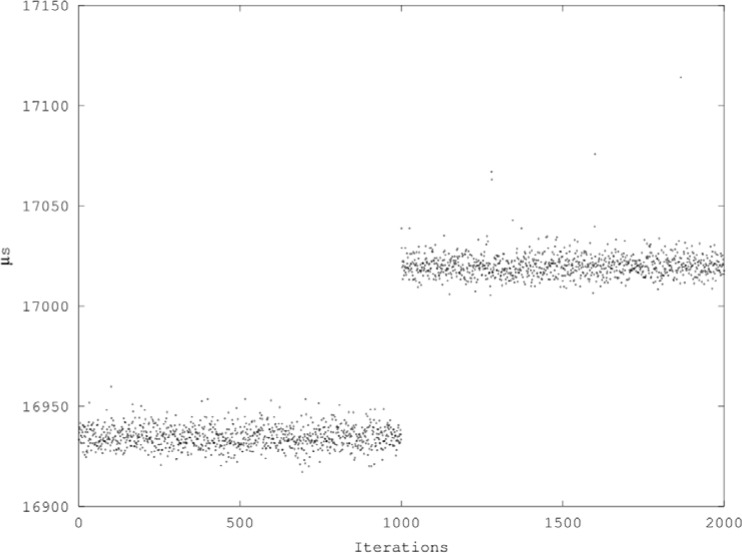
Scatter plot of run 26 data for vector[i] at –O3.

The average measured CPU time for each combination of variant and compiler options was given in Table 4. Review of Table 4 and the other tables revealed the following patterns and anomalies:

- Significant time-dependency or time-varying behavior, as evidenced by Durbin-Watson statistics less than 1, was associated with optimization level –O0 (i.e., the absence of optimization) and with Mudflap.
- To a lesser degree, variants that used index indirection exhibited a pattern of divergence from ideal values in the Durbin-Watson statistics and the ratios of big to small cell means, and their results are difficult to interpret. On the one hand, switching from the unsafe operator[] to the safe at() function while using index indirection did not produce the disproportionate incremental performance degradation that one would have expected to find if the checks in the variants without indirection had been eliminated by optimization. On the other hand, some of the means for vector.at(x[i]) are actually less than the means for comparable unsafe variants that also used index indirection. The mixture of read and write operations in these variants might have caused cache effects and cache-related variability to dominate the results.
- Comparing its mean and Durbin-Watson statistics with those of neighboring cells, it is clear that something anomalous occurred with vector[i] at –O3. The histogram of that cell’s data (Fig. 3)^3^ shows a double peak; there is a main cluster of data peaking at approximately 16 933 *µ*s and a smaller, secondary cluster peaking at approximately 17 019 *µ*s. Scatter plots of individual runs (e.g., Fig. 4) reveal sudden shifts of the mean occurring at different points in the middle of runs.
- In all cases except vector[i] at –O3, the performance of a given variant improved or remained the same to within the margin of error as optimization increased from –O0 to –O3, but in no case did performance improve from the addition of –flto to –O3. (Given the simple structure of the benchmark programs, no benefit from –flto was expected.)
- Any differences in the performance of the same operations compiled as C and C++ with optimization were negligible.

**Fig. 5:**
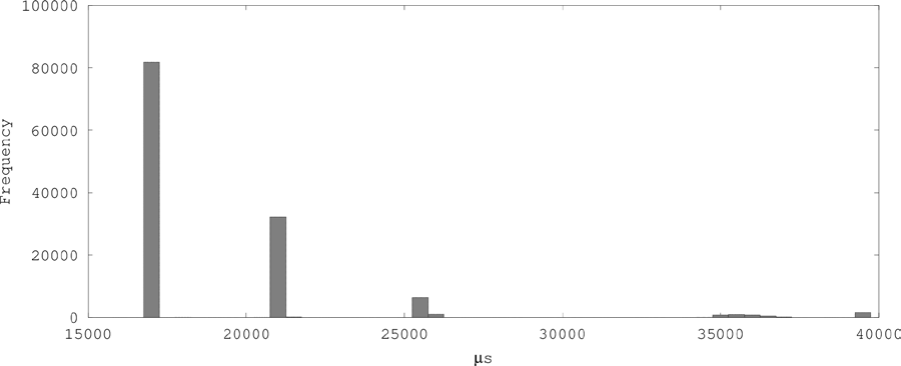
Histogram of cell data for C pointer at –O3 –fmudflap –fmudflapir.

As the Durbin-Watson statistics indicate, Mudflap-enabled runs suffered from significant time-dependencies. The location of the distribution moved by increments on the magnitude of 10^4^ *µ*s, resulting in histograms like Fig. 5. The ratios of big to small cell means reflect a significant amount of added overhead, and inter-process variation was significant as well. However, one benchmark, array[x[i]], particularly with –fmudflapir, showed relatively little impact from Mudflap.

The measurements taken with Mudflap enabled were thus significantly impacted by confounding factors, and a result for the mean performance degradation attributable to Mudflap relative to unchecked C code based on these data would be premature. However, it is possible to observe that Mudflap did not perform better than the other checked approach, vector.at(i). The lowest clusters of data from Mudflap (corresponding to the leftmost bar in Fig. 5) are located higher than the main cluster from vector.at(i) at –O3.

The original paper on Mudflap [30] stated: “Mudflap instrumentation and runtime costs extra time and memory. … When running, it takes time to perform the checks, and memory to represent the object database.” It stated, “The behavior of the application has a strong impact on the run-time slowdown,” and the three applications tested there showed approximate run-time slowdown factors (execution time with Mudflap divided by execution time without Mudflap) of 1.25, 3.5, and 5.

### 4.3 Performance Comparison

The results of interest for comparison are the cell with the best observed performance for safety-checked access versus the cell with the best observed performance overall.

By an insignificant margin, the lowest mean in Table 4 belongs to C array[i] at –O3. In the event that the 12 cells having means in the vicinity of 16 845 *µ*s are actually equivalent, then this choice is as good as any to represent the group. The better-performing safety-checked variant is vector.at(i), for which optimization level –O3 again offers the lowest mean by an insignificant margin.

**Fig. 6:**
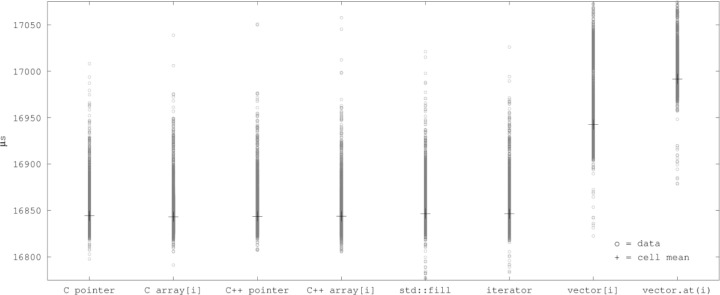
Scatter plot of cell data and means vs. variants at –O3, “big” arrays (outliers not shown).

Figure 6 plots the cell data and cell means for the relevant variants at –O3. The vertical axis of Fig. 6 is zoomed in as much as is reasonable, discarding outliers, yet C pointer, C array[i], C++ pointer, C++ array[i], C++ std::fill, and C++ vector iterator remain nearly indistinguishable.^4^

**Fig. 7:**
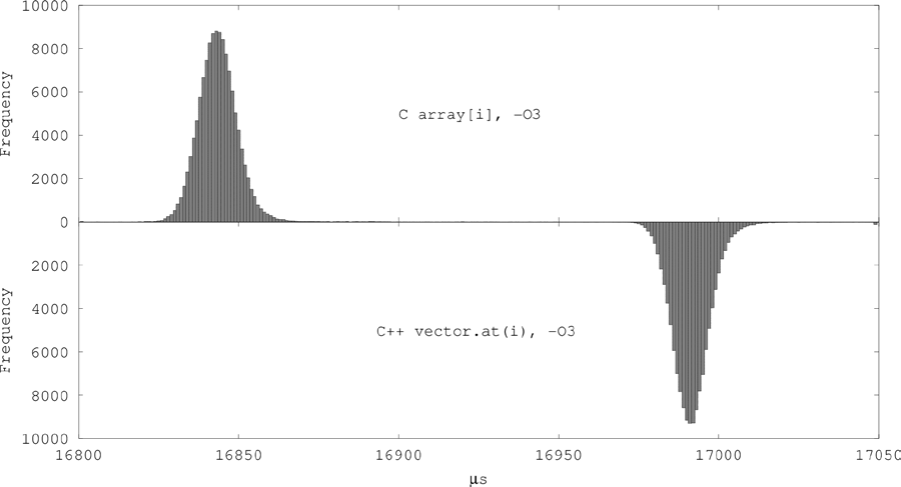
Bihistogram of data for C array[i] vs. C++ vector.at(i) at –O3, “big” arrays.

A bihistogram of the cell data for C array[i] and vector.at(i) at –O3 with all outliers lumped into the first or last bin is shown in Fig. 7.

Computing the relative difference in mean CPU times for these two cells, it follows that the mean relative performance degradation attributable to the use of the at() function instead of C array indexing in this use case is (0.881 ± 0.009) %. The coverage factor for this comparison is computed to be *k* = 4.26 using the Šidák inequality as discussed in Sec. 4.1 to ensure that a coverage of at least 95 % is attained allowing for simultaneous assessment of all cell means and all pairwise comparisons of unsafe array access to safe array access possible in this experiment. The number of effective degrees of freedom associated with the standard uncertainty of this comparison is 75.4, computed using the Welch-Satterthwaite formula [36].

The above result reflects performance of the at() variant relative to the C array variant. However, performance degradation measured in this way is also relative to how much of the program’s entire activity the array accesses represent. In a program that did few array accesses as part of a larger activity, the degradation would be relatively less. The benchmarks used in this study included the overhead of the loops used to traverse the arrays. If the overhead of the loops were measured accurately and then subtracted from the results, the degradation would be relatively greater.

### 4.4 Disassemblies

The basis of the following disassemblies was the output of the command

objdump--disassemble--disassembler-options=×86−64--demangle--no-show-raw-insn

operating on the executables that were used in data collection. Symbols and data mentioned in annotations were identified using the GNU assembler listing obtained by including –Wa,–ahldn=filename.l on the GCC command line.

**Fig. 8:**
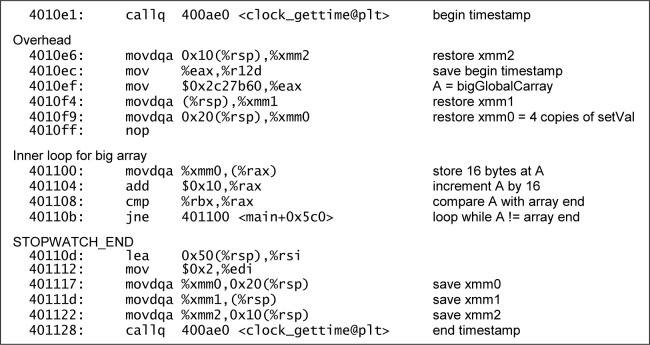
Disassembly of array[i] loop from bench_C_O3.

Figure 8 shows the annotated disassembly for the “big” half of the C array[i] benchmark of bench_C_O3. The assignment of 10^7^ 32-bit values was implemented as a loop that stored 2.5 × 10^6^ 128-bit values.

Interestingly, the implementation of the C pointer benchmark in the same program included instructions to handle the possibility that the size of the array would not evenly divide by 128 bits (which never happened), while the implementation of the array[i] benchmark did not.

**Fig. 9:**
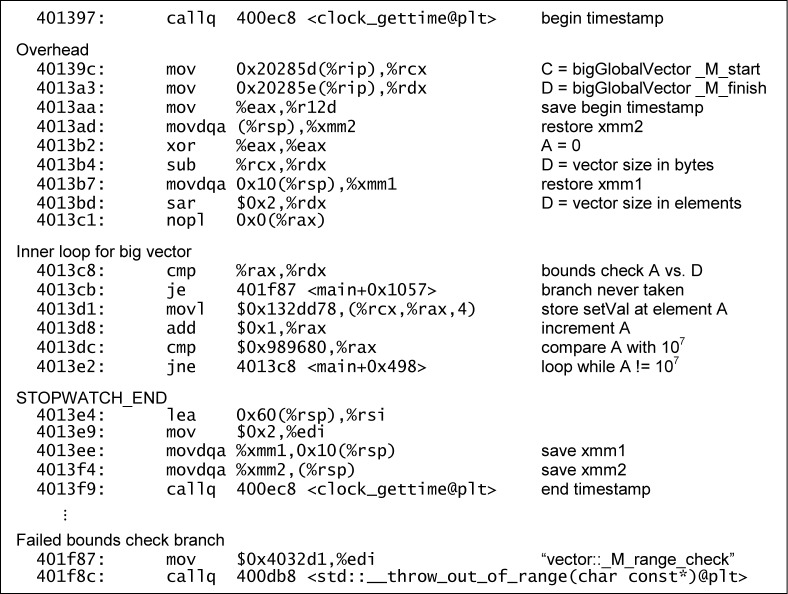
Disassembly of vector.at(i) loop from bench_C++_O3.

Figure 9 shows the annotated disassembly for the “big” half of the C++ vector.at(i) benchmark from bench_C++_O3. Only plain 32-bit assignments were used, so this loop made four times as many iterations as the other. The safety check is present inside the loop, albeit simplified: it was implemented using an equality comparison, rather than a greater-or-equals comparison as specified in the source for at().^5^ This presumably was an optimization for the case where the loop counter only increments by 1.

While both listings include overhead associated with the calls to clock_gettime, neither reflects any instruction reordering or code elimination that would invalidate the results.

**Fig. 10:**
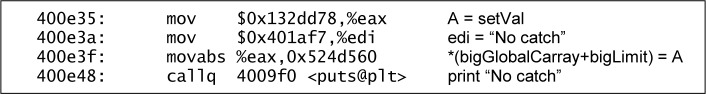
Disassembly of invalid pointer assignment from end of bench_C_O3.

An examination of the end of bench_C_O3 found the out-of-bounds assignment that was flagged by the compiler intact, as shown in Fig. 10.

### 4.5 Robustness of Results

Because many potentially relevant factors were kept constant throughout this study, the results cannot be generalized beyond the case that was tested. Although the observed change in performance is attributable to the change in the array access method, the mechanism by which that code change resulted in a particular performance difference and the robustness of that link were not established. The more counter-intuitive (albeit less reliable) results from the variants that used index indirection suggest that the link is quite fragile.

The impacts of memory bandwidth, cache misses, and similar limiting factors were not measured. Reviewing the results, it is natural to hypothesize that a bottleneck exists somewhere other than the execution of instructions. If such impacts were reduced, the relative difference caused by additional instructions operating on the same data would increase.

The relationships between the data access pattern and the behaviors of the caches—e.g., reads versus writes, size of working set versus sizes of caches, random versus linear traversal, and automatic prefetching—can be expected to have a drastic impact on CPU time. What was a negligible difference in this case could become a times-*X* performance hit if the use case changed to make caching more effective. Additionally, the impact of caching on software performance is so complex that anomalous cases will exist [37,38].

The CPU used in this study had a superscalar architecture with multiple functional units. At the micro-op level, the store operation for a given iteration (or several) probably ran in parallel with the bounds-checking comparison operation for the next iteration (or several) on different functional units. Since the index-out-of-bounds branches were not exercised in the timed regions, branch prediction should have been highly effective.

Other factors that may impact the results are mentioned in the next section as areas to explore in future work.

## 5. Future Work

First priority for future work will be addressing the measurement challenges that this case study encountered: identifying the sources of variability, determining ways to obtain repeatable and reproducible results in the presence of additional complexity such as Mudflap, applying better methods for estimating uncertainty given the complex variance structure of these measurements, and hopefully identifying and controlling the confounding factors whose effects were observed. For example, if measurements are performed in a “bare metal” environment with no interrupts or operating system scheduler activity intervening, how much of the variability goes away? Of course, as more extreme measures are taken to isolate the experiment, the less representative it is of a typical software environment.

Once the measurement challenges have been addressed, exploration of the different dimensions of use cases, hardware configurations, programming languages, and run-time environments can proceed. More combinations of cache sizes, data sizes, and data access patterns should be evaluated, and the same measurements should be performed on a variety of different CPUs, PC configurations, kernel configurations, and compilers, as well as on a variety of different architectures and operating systems, including ARM-based devices with Android or iOS, to determine if the measurement methods and the results are robust across such changes.

The performance of the checked Standard Template Library function could be compared against the performance of analogous operations in a variety of programming languages that normally do check array indices at run time, including Java and C#. Readers have expressed particular interest in results comparing Java code and C code with explicit bounds-checking against the variants already explored.

Any number of different use cases for array-like data structures and other examples of safe and unsafe coding constructs (other than checked and unchecked array indices) could be examined to determine if the results are robust across those changes. One could also focus on the single-iteration performance of an operation immediately after some unrelated workload is executed, rather than on the average performance of an operation that is repeated many times consecutively. A corpus of measurements for simple, synthetic use cases would provide a starting point for estimating the performance impact of including safety checks in larger, more complex applications when direct measurements of that impact are impractical.

If cases are found in which the performance penalty for run-time safety checks is actually sufficient to justify the use of unsafe coding constructs, the relative performance of parallelized versions of the benchmarks using multiple CPU cores could then be examined. If the additional load created by run-time safety checking could be shifted onto underutilized resources without an excessive amount of synchronization overhead, the significance of that load might be reduced to a question of energy efficiency rather than execution speed.

## 6. Conclusion

The observed mean performance degradation attributable to use of the at() member function of the C++ Standard Template Library’s vector class instead of an unchecked array assignment was only (0.881 ± 0.009) %. While the performance degradation is expected to vary in different use cases, the simple array traversal that was tested here is a fundamental pattern of basic programming. These results suggest that the performance benefits of unsafe methods of accessing arrays in C and C++ can be negligible when weighed against the negative consequences for security and software quality that are described in Ref. [7]. Comparisons across different use cases, configurations, programming languages, and environments are needed to determine under what circumstances (if any) the performance advantage of unchecked access is actually sufficient to outweigh the negative consequences for security and software quality.

The raw data from this case study are available online [39].

## Figures and Tables

**Table 1:** Hardware and software environment

PC model	Dell Precision T5400
CPUs	2 × Xeon X5450 quad core (dual die) 3 GHz (S-Spec SLASB)
L1 cache	32 KiB instruction cache + 32 KiB write-back data cache per core, 64 sets, 8-way associative, 64 B coherency line size
L2 cache	2 × 6 MiB unified cache per CPU (6 MiB per die, shared by 2 cores), 4096 sets, 24-way associative, 64 B coherency line size
L3 cache	No

RAM	8 × 1 GiB DDR2-667D ECC FB-DIMM
BIOS	A07 2010-12-09
SpeedStep	off
Snoop filter	on
Adjacent cache line prefetch	off
Hardware prefetch	on

Base environment	Slackware 13.37 64-bit
Kernel	Linux 3.2.2 (mainline)
Clocksource	TSC
Timer frequency	1000 Hz
Tickless system (dynamic ticks)	yes
Accelerate last non-dyntick-idle CPU’s grace periods	yes
Preemption model	No forced preemption (server)
Transparent hugepage support	yes / always
CPU frequency scaling	no
CPU idle PM support	yes
Cpuidle driver for Intel processors	no
Compiler	GCC 4.6.2

**Table 2:** List of variants

Variant	Code sample
C pointer	*iptr = setVal;
C array[i]	bigGlobalCarray[i] = setVal;
C array[x[i]]	bigGlobalCarray[indices[i]] = setVal;
C++ pointer	*iptr = setVal;
C++ array[i]	bigGlobalCarray[i] = setVal;
C++ array[x[i]]	bigGlobalCarray[indices[i]] = setVal;
C++ vector std::fill	std::fill (bigGlobalVector.begin(), bigGlobalVector.end(), setVal);
C++ vector iterator	*it = setVal;
C++ vector[i]	bigGlobalVector[i] = setVal;
C++ vector.at(i)	bigGlobalVector.at(i) = setVal;
C++ vector[x[i]]	bigGlobalVector[indices[i]] = setVal;
C++ vector.at(x[i])	bigGlobalVector.at(indices[i]) = setVal;

**Table 3:** Executables

File name	Size (B)	File name	Size (B)
bench_C_O0	21 889	bench_C++_O0	71 094
bench_C_O1	25 522	bench_C++_O1	63 806
bench_C_O2	25 834	bench_C++_O2	62 382
bench_C_O3	27 570	bench_C++_O3	76 518
bench_C_O4	25 676	bench_C++_O4	38 846
bench_C_O5	34 392		
bench_C_O6	35 431		

**Table 4:** Cell means with expanded uncertainty (*µ*s), “big” arrays (italics where Durbin-Watson < 1)

	C pointer	C array[i]	C array[x[i]]	C++ pointer	C++ array[i]	C++ array[x[i]]
–O0	*24 935.1* ± *260.5*	*25 999.1* ± *190.6*	*45 438.0* ± *4081.3*	*26 686.7* ± *259.0*	*27 556.0* ± *150.4*	*32 053.0* ± *166.1*
–O1	16 924.6 ± 0.4	16 924.6 ± 0.4	27 776.4 ± 1.4	16 926.5 ± 1.2	16 925.8 ± 1.0	27 776.5 ± 4.7
–O2	16 924.2 ± 0.4	16 924.4 ± 0.3	27 769.3 ± 1.7	16 925.2 ± 1.1	16 925.5 ± 1.0	27 770.6 ± 4.9
–O3	16 844.3 ± 0.5	16 843.3 ± 0.5	27 770.3 ± 1.9	16 843.7 ± 0.7	16 843.7 ± 0.5	27 767.6 ± 4.3
–O3 –flto	16 844.0 ± 0.4	16 843.4 ± 0.4	27 769.7 ± 1.4	16 844.3 ± 1.0	16 844.3 ± 0.8	27 770.1 ± 4.7
–O3 –fmudflap –fmudflapir	*19 462.2* ± *1341.5*	*21 140.8* ± *1320.9*	28 179.8 ± 13.1			
–O3 –fmudflap	*21 302.9* ± *1393.7*	*20 494.0* ± *1169.1*	*35 794.8* ± *85.6*			

	C++ vector std::fill	C++ vector iterator	C++ vector[i]	C++ vector.at(i)	C++ vector[x[i]]	C++ vector.at(x[i])
–O0	*26 511.9* ± *163.2*	*189 772.8* ± *865.1*	*49 996.7* ± *530.3*	*138 742.2* ± *5770.1*	*84 470.7* ± *536.7*	*145 592.4* ± *6781.9*
–O1	16 932.2 ± 2.5	16 953.7 ± 3.8	17 025.1 ± 1.5	21 224.2 ± 1.7	27 781.1 ± 7.1	28 059.6 ± 6.9
–O2	16 932.4 ± 2.3	16 932.5 ± 2.3	16 932.9 ± 2.3	16 991.9 ± 1.6	27 743.9 ± 7.3	27 715.8 ± 6.9
–O3	16 846.6 ± 0.8	16 846.4 ± 1.4	*16 942.6* ± *12.7*	16 991.7 ± 1.4	27 521.6 ± 7.6	27 496.6 ± 7.1
–O3 –flto	16 846.3 ± 1.0	16 846.4 ± 1.2	16 932.3 ± 2.9	16 992.4 ± 2.2	*27 975.5* ± *7.8*	27 846.6 ± 8.5

**Table 5:** Cell Durbin-Watson statistics, “big” arrays

	C pointer	C array[i]	C array[x[i]]	C++ pointer	C++ array[i]	C++ array[x[i]]	C++ vector std::fill	C++ vector iterator	C++ vector[i]	C++ vector.at(i)	C++ vector[x[i]]	C++ vector.at(x[i])
–O0	0.1	0.2	0.0	0.3	0.1	0.0	0.1	0.0	0.0	0.0	0.0	0.0
–O1	1.9	1.9	1.9	1.7	1.7	1.4	2.0	1.9	2.0	1.9	1.3	1.2
–O2	1.9	1.9	1.9	1.7	1.7	1.3	2.0	2.0	1.9	2.0	1.1	1.2
–O3	1.9	2.0	1.9	2.0	2.0	1.4	2.1	2.0	0.6	2.0	1.2	1.2
–O3 –flto	1.9	2.0	1.9	2.0	2.0	1.4	2.1	2.0	1.9	2.0	1.0	1.1
–O3 –fmudflap –fmudflapir	0.1	0.1	1.6									
–O3 –fmudflap	0.1	0.1	0.7									

**Table 6:** Ratios of cell means, big/small

	C pointer	C array[i]	C array[x[i]]	C++ pointer	C++ array[i]	C++ array[x[i]]	C++ vector std::fill	C++ vector iterator	C++ vector[i]	C++ vector.at(i)	C++ vector[x[i]]	C++ vector.at(x[i])
–O0	10.0	10.0	10.1	10.0	10.0	10.0	10.0	10.0	9.9	10.1	9.7	10.1
–O1	10.0	10.0	9.8	10.0	10.0	9.8	10.0	9.9	9.9	10.0	9.8	9.8
–O2	10.0	10.0	9.8	10.0	10.0	9.8	10.0	10.0	10.0	10.0	9.8	9.9
–O3	10.0	10.0	9.8	10.0	10.0	9.8	10.0	10.0	10.0	10.0	9.8	9.9
–O3 –flto	10.0	10.0	9.8	10.0	10.0	9.8	10.0	10.0	9.9	10.0	9.9	9.9
–O3 –fmudflap –fmudflapir	4.7	3.7	10.0									
–O3 –fmudflap	3.6	4.1	10.0									

**Table 7:** Ratio of the experimental standard deviation of the mean of series means to the experimental standard deviation of the mean of all cell data

	C pointer	C array[i]	C array[x[i]]	C++ pointer	C++ array[i]	C++ array[x[i]]	C++ vector std::fill	C++ vector iterator	C++ vector[i]	C++ vector.at(i)	C++ vector[x[i]]	C++ vector.at(x[i])
–O0	43.8	42.8	45.1	42.1	43.7	44.6	43.5	45.0	45.0	45.1	45.1	45.1
–O1	4.5	4.9	6.4	13.0	12.3	24.6	7.3	13.4	5.8	8.5	26.8	27.1
–O2	4.5	4.5	7.6	11.9	12.4	25.6	7.0	10.1	11.8	8.7	28.8	27.2
–O3	1.6	1.5	8.6	1.6	2.7	23.6	3.0	6.5	31.5	6.4	27.3	28.6
–O3 –flto	1.3	1.2	6.5	2.3	4.3	24.8	3.7	5.2	13.5	9.9	31.6	29.1
–O3 –fmudflap –fmudflapir	20.1	19.2	19.4									
–O3 –fmudflap	19.2	20.7	5.8									

**Table 8:** Outcomes of attempted out-of-bounds assignments

	C pointer	C++ pointer	vector.at(x[i])
–O0	No catch	No catch	std::out_of_range
–O1	No catch	No catch	std::out_of_range
–O2	Compiler warning, no catch	Compiler warning, no catch	std::out_of_range
–O3	Compiler warning, no catch	Compiler warning, no catch	std::out_of_range
–O3 –flto	Compiler warning, no catch	Compiler warning, no catch	std::out_of_range
–O3 –fmudflap –fmudflapir	Compiler warning, aborted		
–O3 –fmudflap	Compiler warning, aborted		

## Footnotes

1 Specific computer hardware and software products are identified in this report to support reproducibility of results. Such identification does not imply recommendation or endorsement by the National Institute of Standards and Technology, nor does it imply that the products identified are necessarily the best available for the purpose.

2 Additionally, the GNU Compiler Collection (GCC)’s implementation of Java allows bounds checking on array indices to be turned off with –fno-bounds-check [5], but this is not a standard Java language feature.

3 The convention in all of the histograms is for any data lying outside the range of bins selected for the plot to be lumped in with the first or last bin; hence, the entire weight of the long tail is reflected in the rightmost bin.

4 The std::fill algorithm takes parameters to identify the beginning and end of the range to be filled. In the stl_algobase.h header file that came with GCC 4.6.2, it is implemented as an iterator assignment loop similar to the C++ vector iterator variant where individual assignments are unchecked. If _GLIBCXX_DEBUG is defined, some form of validation is performed on the supplied iterators. However, that option is off by default and was not enabled for the programs used here. Consequently, std::fill is categorized as an unchecked access method.

5 The at() function, defined in the stl_vector.h header file that came with GCC 4.6.2, throws an exception if the condition (__n >= this–> size()) is true. A lower bound would be redundant because the data type of the index is unsigned.
